# Development and Evaluation of Fall Impact Protection Pads Using Additive Manufacturing

**DOI:** 10.3390/ma12203440

**Published:** 2019-10-21

**Authors:** Jung Hyun Park, Hee-Kyeong Jung, Jeong Ran Lee

**Affiliations:** Department of Clothing and Textiles, Pusan National University, Busan 46241, Korea; jhpark1391@pusan.ac.kr (J.H.P.); jhkfashion@pusan.ac.kr (H.-K.J.)

**Keywords:** additive manufacturing, protection pad, protective pants, fall-impact protection, design

## Abstract

This paper presents the development and evaluation of fall-impact protection pants for elderly women using additive manufacturing. The protective pants were designed incorporating a protective pad in the hip area to reduce the impact of falls on the human body. The protective pad is a 3D mesh structure with a curved surface to fit the human body. Pads printed with flexible thermoplastic polyurethane were combined with foam to create the final pad. The impact-absorbing performance of the pad was verified through physical impact experiments. When dropping a bowling ball onto the protective pad from heights of 15, 20, and 25 cm, the protective pad was found to reduce the impact force by more than 82% in all cases. The impact force was less than the average fracture threshold of 3472 N. A subject group and an expert group evaluated the appearance, pad characteristics, motion functionality, and the wearability of the protection pants. Despite the insertion of a pad, the pants appeared natural and had a good fit. The pads were evaluated as being well-designed in terms of their position, shape, area, thickness, weight, flexibility, ease of insertion, and ease of use. Users were comfortable performing various motions when wearing the designed protective clothing. Therefore, this work can be considered to have developed protective clothing that provides satisfactory impact-protection performance and comfort thereby advancing the possibility of applying additive manufacturing to the creation of functional garments.

## 1. Introduction

The additive manufacturing industry has been expanding its scope of application [1], from the manufacturing industry to the biomedical [2,3,4], agricultural [5], composites [6], aerospace [7,8], and fashion industries [9,10], as a next-generation growth engine that will lead to industrial innovation and creative economy vitalization [11]. The additive manufacturing market is growing explosively because of the development of related technologies and the efforts of companies to innovate and catch up with the rapidly changing tastes of consumers. In the field of fashion, additive manufacturing is mainly used for the development of accessories such as shoes and hats because of relatively easy production [12,13]. In the case of apparel and textiles, aesthetic characteristics are usually emphasized although practicality is limited [14,15]. Improving the usability of additive-manufacturing fashion products and utilizing the advantages of the custom-made 3D shapes can expand the additive manufacturing fashion field and contribute to practical use. In medical and virtual garment fields, 3D scanning technology has also been used for capturing patient/customer dimensions [16,17].

The elderly have poorer balance and a lower ability to deal with accidents when compared to those of younger people and, therefore, are easily injured by falls. Older people suffer from greater degrees of injury when they fall, owing to their weak muscles and bones. In addition, the elderly who have experienced falls are not only dependent on others but also suffer from psychological difficulties, such as depression and/or loss of confidence [18]. As fractures caused by falls can be physically and psychologically fatal to the health and quality of life of the elderly, it is important to prevent fractures. Hip protectors are effective in reducing the risk of hip fractures by attenuating the impact of a fall [19,20]. However, it has been reported that elderly people are reluctant to wear hip protectors because of the inconvenience, difficulty in wearing and removing them, and deterioration of appearance [21,22]. Therefore, it is important to improve the appearance and convenience of use as well as the protective effect, in order to increase the utilization of the hip protector.

So far, most of the experimental studies on hip protectors have been conducted from a biomechanical perspective. In order to verify the effectiveness of the hip protector, experiments using testing simulators were performed under various conditions such as different physical properties, thicknesses, and positions of the pads, different falling angles, and various flooring materials [23,24,25]. Research on analyzing the impact force through finite-element modeling was also conducted to utilize the information, during the development of hip-protection devices [26,27]. However, it is difficult to find studies that suggest improvements for hip-protection devices, in terms of the wearing characteristics and protective effect. Accordingly, it has become necessary to develop a hip-protection device with high suitability and utilization, which reflects the human body shape.

The purpose of this study was to develop impact-protection pants with curved 3D-mesh pads, using additive manufacturing to verify the impact-protection performance through physical experiments and to evaluate the appearance, pad characteristics, motion functionality, and wearing characteristics of the pants. In this study, the curved 3D mesh pads were designed to develop the impact protection pads with shapes adaptive to the human body surface, and its functionality was verified by presenting the results of the wearing sensory quality evaluation tests. Since there are a limited number of studies on the development of fabric with curved mesh structure, it was necessary to utilize additive manufacturing to overcome such a problem. Additive manufacturing can be applied to develop a protective pad with the desired properties and shapes by adjusting the conditions of detailed additive parameters. Additionally, we used body scan data for the 3D modelling of the pad and which allowed for the design of complex three-dimensional structures reflecting the curved surface of the human body in order to improve the fit and wearing comfort. We targeted for over 60 female subjects to design and develop the pads using the average body scan data of over 60 women. We expected that it was possible to apply the developing processes of this study to other body types, sizes, and ages by utilizing additive manufacturing characteristics for a small quantity of customized productions effectively.

## 2. Materials and Methods

### 2.1. Design of Protective Pants

The pants were designed to reduce the impact of falling by inserting a protective pad in the hip area. In this work, the impact-protection pants were designed to be worn by older women performing daily-life activities and were developed with an emphasis on impact-protection performance, motion functionality, and ease of use. 

The impact transmitted in the vertical direction when falling backwards can cause compression fractures in the spine; therefore, it is necessary to protect the bottom of the hip. Hip fractures are caused by impacts to the great trochanter, a bone that protrudes from the upper part of the femur, and most of such fractures require surgery and long hospitalization which increases the risk of death. Therefore, protecting the sides of the hip is also necessary.

**Figure 1:**
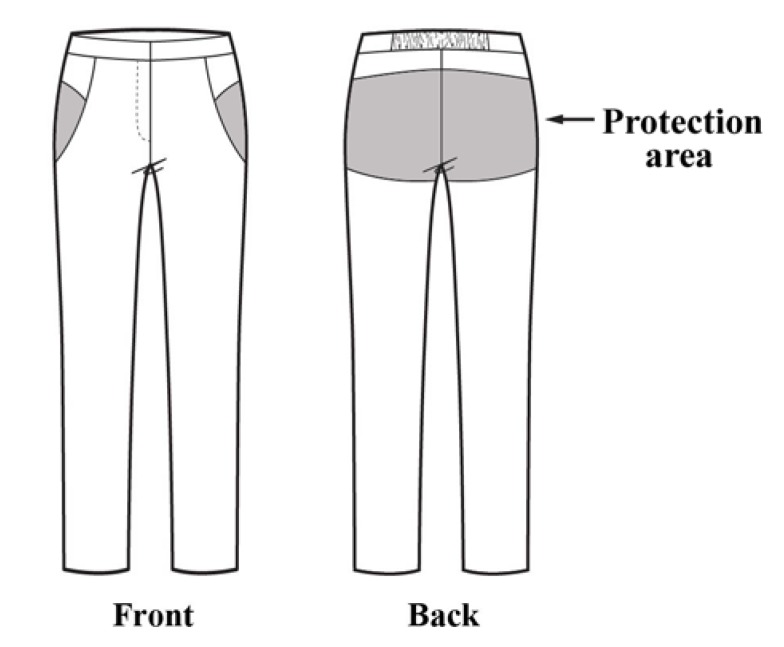
Design of the protective pants. Protection area is shaded.

In our design, a pocket was made by inserting the designed cutting lines at the edges of the protective part and placing the lining. The design was based on the outer pocket method so that the protective pad could be inserted and removed from the outside easily, even while wearing the pants. The designed cutting lines fixed the pad position while providing an optical illusion wherein the difference in thickness due to the pad insertion was not noticeable. The pants had a slim straight-fit silhouette, which is preferred by older women, and an elastic band was inserted into the back of the waist belt to prevent the pants from loosening and to improve the fit (Figure 1).

### 2.2. Pattern Design

**Figure 2:**
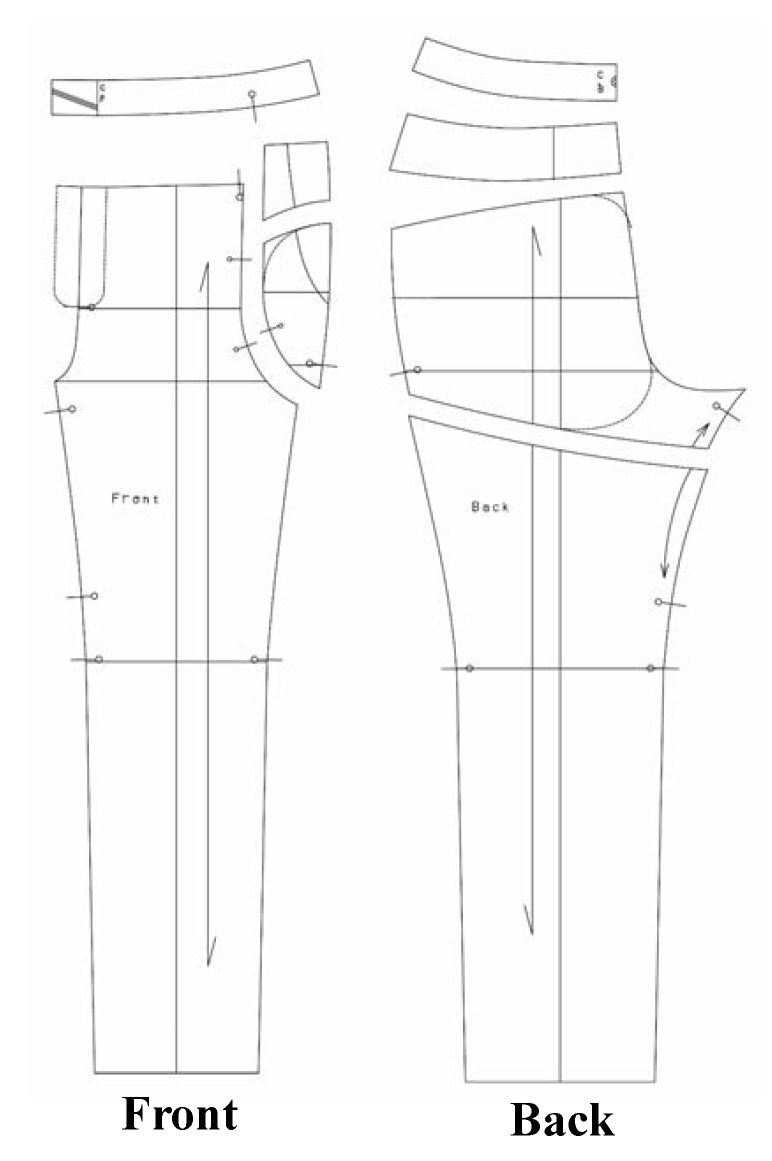
Pattern design for protective pants.

**Figure 3:**
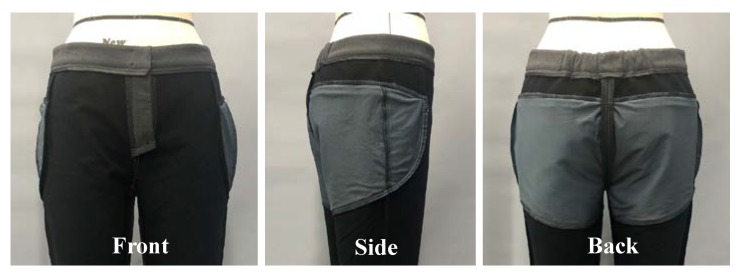
Pocket construction inside the pants.

The pants’ pattern was designed considering the physical characteristics and motion functionality of elderly women. The body measurements for the pattern design were set as follows: waist circumference of 82 cm, hip circumference of 92 cm, hip length of 20 cm, body rise of 26 cm, and pants length of 95 cm, referring to the average size of women, calculated in the 7th South Korea Human Size Survey. The waist belt used an elastic band of length 20 cm (in total) with 10 cm on either side of the center back line. As shown in Figure 2, the pattern was cut by drawing a design line on the edge of the protective part and creating a lining pattern. To evaluate the properties of the fabrics, the standard test methods of KS K 0210 (test methods for quantitative analysis of fiber mixtures of textiles), KS K 0514 (measuring method for the weight of cloth), KS K ISO 5084 (measuring method for the weight of cloth), and KS K 0352 (test method for the stretch properties of stretch woven fabrics) were used to determine the fiber content, weight, thickness, and stretch of the fabrics, respectively. As shown in Table 1, the outer material of the pants was a lightweight and stretchable functional fabric and the lining was composed of stretchy and breathable mesh fabric. The pockets were created by placing the lining according to the protection area (Figure 3), and the opening was created by attaching an invisible zipper.

### 2.3. Additive Manufacturing of Pad and Integration

**Figure 4:**
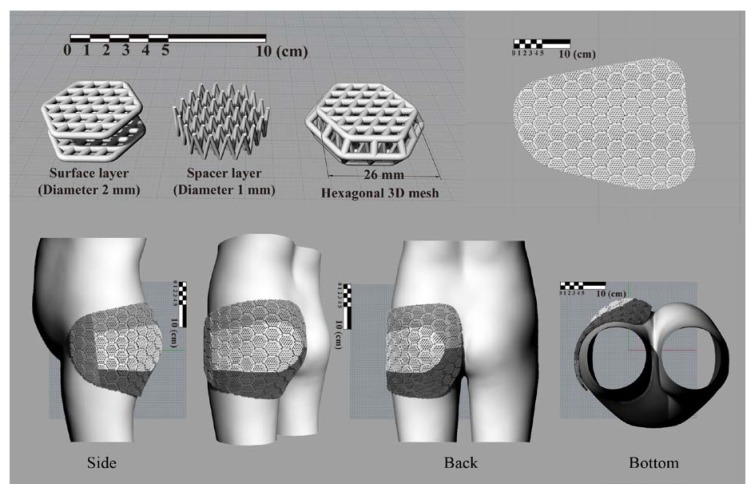
3D modeling of curved pad.

The protective pad was modeled using the Rhinoceros 5.0 software (Robert McNeel & Associates, Seattle, WA, USA). The basic unit of the pad was a 3D hexagonal mesh, which had impact-absorbing properties because it was composed of a surface layer (diamond type) and a spacer layer (V-shape). The basic units were connected to each other in a honeycomb shape to form a flexible mesh structure. The mesh structure was deformed according to the body’s curvature to improve the fit. As it was difficult to print the entire pad at once, the pad was divided into four pieces, considering the position of the side lines and the shape of the surface (Figure 4). 

The 3D model, saved as a stereolithography (STL) file, was converted to a file compatible with 3D printers by generating the G-code using Cubicreator 2.5.2 (CUBICON Inc., Seongnam-si, Korea). The pads were printed at a temperature of 230 °C, using a flexible thermoplastic polyurethane (TPU) filament, NinjaFlex^®^ (NinjaTek, Manheim, PA, USA), by a Cubicon Single 3D printer (CUBICON Inc., Seongnam-si, Korea) based on the fused filament fabrication (FFF) method. Since process parameters have a remarkable effect on the properties of additive manufacturing parts [28], the specifications of TPU filaments and the conditions of additive manufacturing are shown in Table 2 and Table 3, respectively. The excellent flexibility of TPU contributes to the easy motion of the pads as well as alleviates the impact from falling. The materials consumption in additive manufacturing is also shown in Table 4. The material extrusion method was selected due to the fact of its good flexibility and relatively low manufacturing costs. We estimated the cost of printing the pads as USD $38 in this study.

**Figure 5:**
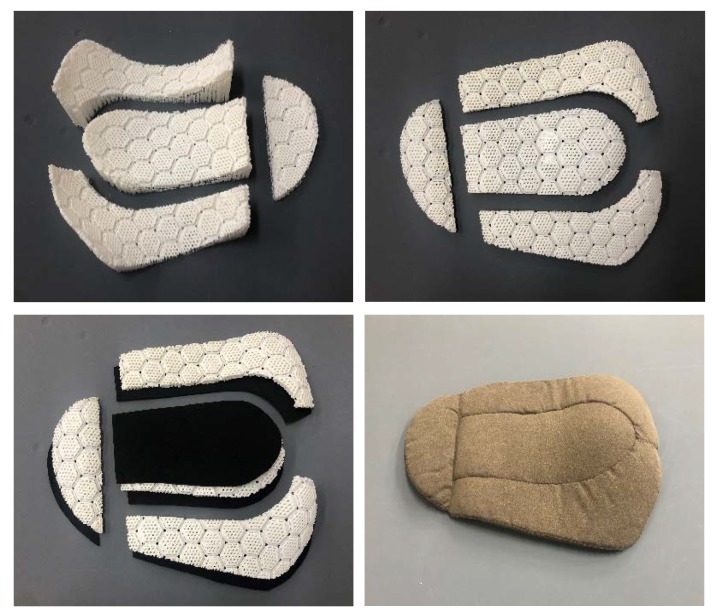
Composition of a 3D-printed curved pad.

After the supporters were removed from the printed four-piece pads, a 2 mm thick layer of chloroprene rubber foam (CR foam) was added to the surface that touched the human body, in consideration of wearing comfort, and a 1 mm thick foam was added to the outer side of the centeral piece for improving the appearance (Figure 5). Properties of CR foam are shown in Table 5. Each component was inserted into a pad case made of stretchable fabric and fixed to function as a single pad (Figure 5), and the connected pad was inserted into a pocket of the pants to be fixed in a protective position.

### 2.4. Evaluation of the Impact-Protection Performance

**Figure 6:**
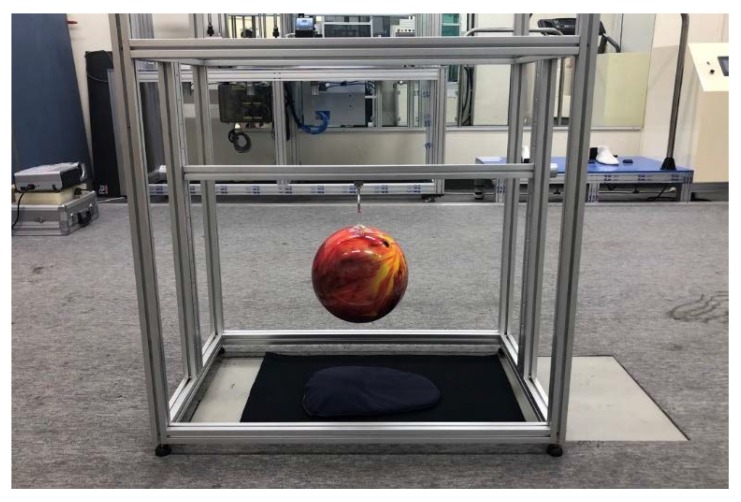
Picture of the impact performance test equipment.

To evaluate the impact-protection performance, drop test equipment were arranged as shown in Figure 6. After installing the support on a force plate, a six-pound bowling ball was dropped from various heights on to the protective pad. The impact value generated vertically when falling was measured and compared with the impact value without the pad. The test was repeated 35 times, at heights of 15, 20, and 25 cm. The impact values without pads at heights of 15, 20, and 25 cm were calculated through extrapolations from force values measured at heights of 6, 9, and 12 cm, because the force plate used in this study was limited to measuring forces above 5500 N. To prevent damages to the sensor and force plate, a 6 mm thick neoprene fabric was laid on the force plate.

### 2.5. Wearing Evaluation 

The subjects were recruited based on the statistical information of 60 women’s body sizes referred by the 7th South Korea Human Size Survey (2015). The number of subjects was 16, including three professional fitting models. The average age of the 16 subjects was 71 ± 4.63 years old. The measured body size of the subjects is shown in Table 6. The wearing evaluation of the impact-protection pants was conducted by a subject group comprising 13 women and an expert group comprising 10 professionals with postgraduate degrees in clothing. The subjects evaluated the appearance of the designed pants by looking in the mirror while wearing the pants. They evaluated their motion functionality after performing six types of movements performed frequently in daily life. The pad characteristics and wearing characteristics were evaluated comprehensively, in terms of practicality, when wearing the pants. The expert group evaluated the appearance by observing the fronts, sides, and backs of three professional fitting models wearing the pants and the pad characteristics by examining the actual pants and manipulating the pads. A 5 point Likert scale, interval scale, was used as the evaluation scale for all parameters, where “1 point” meant “strongly disagree” and “5 points” meant “strongly agree”. Descriptive statistics were used to evaluate the sensory quality of the appearance, pad characteristics, motion functionality, and wearing characteristics. The Mann–Whitney U test was performed to compare the results on the appearance evaluation and pad characteristics between subjects and the expert group.

## 3. Results and Discussion

### 3.1. Evaluation of the Impact-Protection Performance 

**Figure 7:**
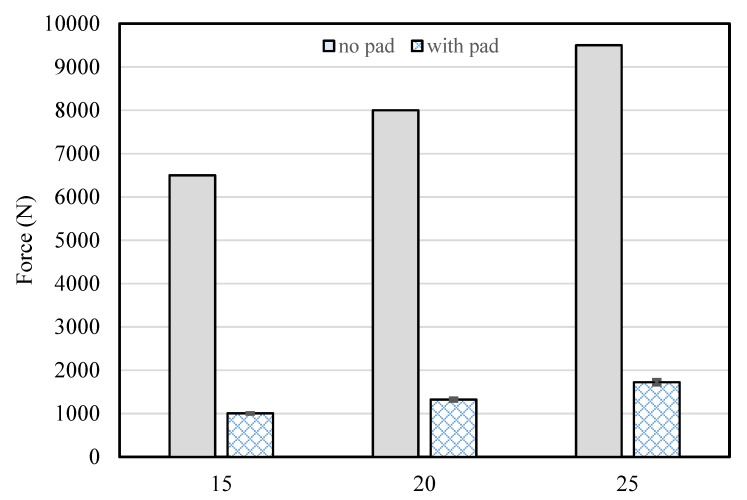
Impact-protection performance test without pad and with pad, in the cases of application of forces generated at heights of 15, 20, and 25 cm.

When a force of approximately 6500 N was applied to the pad from a height of 15 cm, an impact value of 1005 ± 16 N was obtained with the protective pad indicating a decrease of 84.5%. When a force of approximately 8000 N was generated from a height of 20 cm, an impact value of 1312 ± 35 N was observed with the protective pad representing an impact reduction of 83.6%. When approximately 9500 N was applied from a height of 25 cm, an impact value of 1723 ± 61 N was obtained with the protective pad, and a decrease of 81.9% was achieved, as shown in Figure 7. In general, the maximum impact value on the pelvis during a fall is known to be approximately 8000 N. The impact-protection pants used in this study were capable of reducing the impact to below the average fracture threshold of 3472 N.

**Figure 8:**
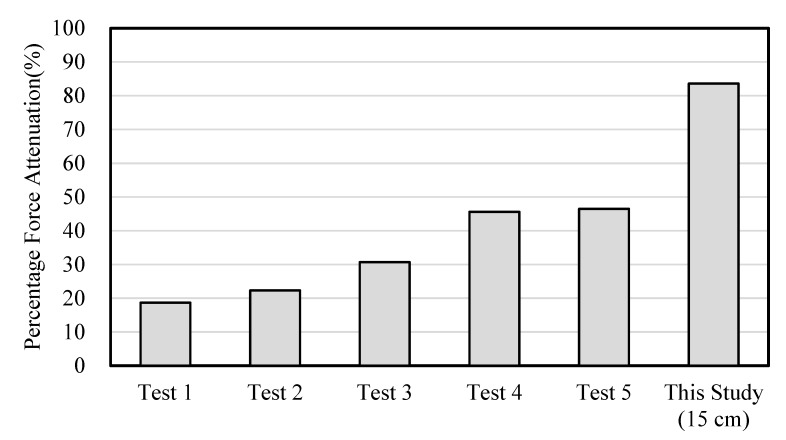
Percentage force attenuations. Tests 1, 2, 3, 4, and 5 were conducted with Safety Pants (Raunomo Oy, Tampere, Finland), Gerihip (Pervent Products, Inc., Rochester, MN, USA), Lyds Hip Protector (Lyds International BV, Velsen-Noord, The Netherlands), HipSaver (HipSaver, Inc., Norwood, MA, USA), and Safety Pants (Van Heek Medical, Venray, The Netherlands), respectively [25].

Percentage force attenuation (%) was calculated to obtain the impact reduction performance of the curved three-dimensional mesh protection pad using Equation (1).

(1) $$\mathrm{Percentage}\;\mathrm{Force}\;\mathrm{Attenuation}\;\left(\%\right)=\left(1-\frac{F_{T}}{F_{0}}\right)\times 100$$

where *F_T_* is the impact force (N) using the protection pad and *F*_0_ is the impact force (N) without the protection pad, respectively. We achieved a percentage force attenuation (%) of 83.6% with the drop height of 15 cm. As shown in Figure 8, Schoor et al. [25] reported the percentage force attenuations (%) with a range of 18.7–46.5% using commercially available soft hip protectors, such as polyurethane- and polystyrene elastomer-based hip protectors. Commercially available soft hip protector pads were tested by applying the force of 6378 ± 141 N using a drop impact test with a substitute pelvis and 0.5 inch thick soft tissue. Thus, the results of the percentage force attenuation indicated that the curved three-dimensional mesh protection pad provided excellent impact protection performance. 

### 3.2. Evaluation of Appearance

**Figure 9:**
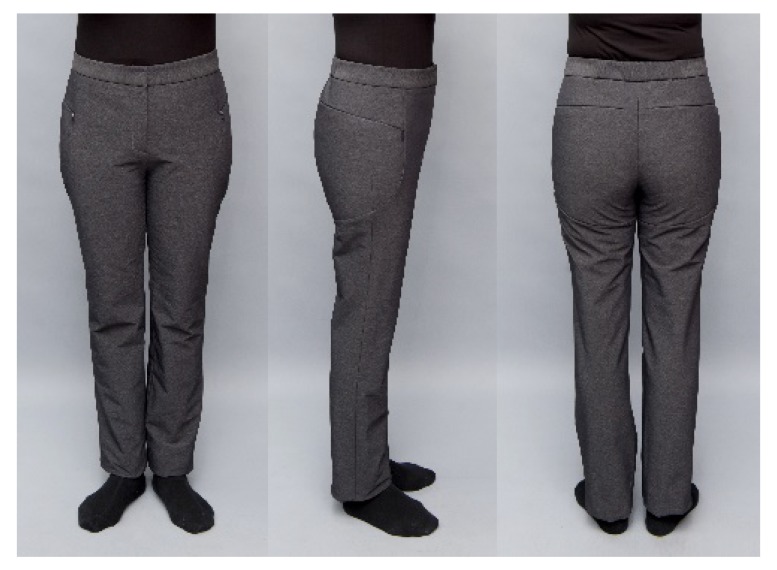
Appearance of the designed pants.

For parameters related to the appearance evaluation (Figure 9 and Table 7), the subject group awarded 4.69 points or higher and the expert group awarded 4.00 points or higher. Both the subjects and experts were satisfied with the color and material of the pants because dark gray, which was preferred by the elderly women, was selected and a material with good elasticity and functionality was used. The design line at the part where the pad was inserted was identified properly, in consideration of the protective parts, and even though a 13 mm thick pad was inserted, the part where the pad was inserted looked natural. Because of the proper consideration given to the stretch of fabric and the pad thickness, the fit at the waist circumference and hip circumference was good, without wrinkles/tugging at the crotch area.

The Mann–Whitney’s U-test was performed to compare the appearance evaluation between subjects and the expert group. As shown in Table 7, there were differences (*p* < 0.05) in the appearance evaluation for color, fit, design line, hip circumference, and crotch area contents between subjects and the expert group. The difference of fit content was especially significant between subjects and the expert group, because the expert group evaluated the appearance by observing the subject models, while the subject group responded to the question while wearing the pants. Therefore, we assumed that the subject group showed better satisfaction on the fit evaluation than the expert group.

### 3.3. Evaluation of Pad Characteristics

**Figure 10:**
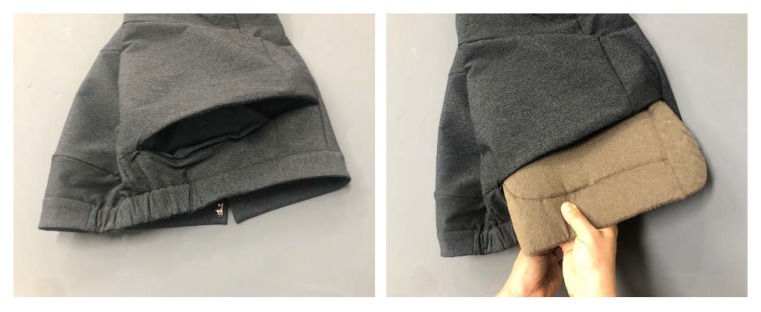
Insertion and removal of pad.

The pad characteristics were evaluated in terms of their position, form, area, thickness, weight, flexibility, insertion method, and ease of use. As shown in Table 8, a high score of four or more was awarded for the shape, area, and position of the pad, because the pad was designed to cover the hips and pelvis, considering appearance and protection. In the expert evaluation, a score of 3.90 was awarded for the thickness of the pad, which was slightly lower than the scores for other parameters. However, in the subject evaluation, a score of 4.69 was awarded, indicating that the thickness of the pad was sufficiently comfortable during actual wearing. The weight of the pad was awarded 4.00 points or higher in both the subject and expert evaluations, confirming that the pads were designed to be within the range of weight that could be worn reasonably. Thickening the intensive protection area and thinning toward the edges reduced the inconvenience caused by the thickness and weight of the pad. The flexibility of the pads showed good results because the pads were designed to be flexible in terms of the structure and were printed using a flexible TPU. The subject group gave a perfect score or near-perfect score for the pad insertion method and ease of manipulation, and the expert group gave a good score of 4.50 or higher. This may be because the pocket is fixed securely and the pads can be inserted and removed easily from the outside opening, as shown in Figure 10.

The Mann–Whitney’s U-test was performed to compare the evaluation of pad characteristics between subjects and the expert group. As shown in Table 8, there were differences (*p* < 0.05) in thickness, flexibility, and pad insertion method contents. Since the average scores from the subject group were higher in the evaluation of pad characteristic for thickness, flexibility, and pad insertion method contents than the expert group, the experts tended to give a slightly lower score than the subject group.

### 3.4. Evaluation of Motion Functionality 

The subject group evaluated the motion functionality after performing various movements while wearing the protective clothing. As shown in Table 9, all parameters scored 4.69 or higher. Therefore, even when wearing protective clothing with a pad, there was no inconvenience in movement. In particular, the subjects did not feel uncomfortable when sitting on a chair or bending their upper body. The pad was designed to fit the curved surfaces of the human body and had flexibility, which contributed to good motion functionality. We calculated the Cronbach’s alpha for determining the reliability among the contents. The Cronbach’s alpha is a measure of internal consistency, that is, how closely related a set of items are as a group. We obtained the Cronbach’s alpha of 0.62 indicating acceptable reliability of contents in the evaluation of motion functionality.

### 3.5. Evaluation of Wearing Characteristics 

As shown in Table 10, a score of 4.77 or higher was rated for all aspects of the wearing characteristics for the pants. The stretch and feel of the pants were good, indicating that the choice of fabric was appropriate. It was convenient to put on and take off the pants even when the protective pads were inserted. No discomfort or pressure was felt at the sites where the impact-protection pads were inserted and, therefore, the design and insertion of the pads were deemed appropriate, considering the characteristics of the human body. We obtained the Cronbach’s alpha of 0.61 indicating acceptable reliability of the contents in the evaluation of wearing characteristics.

## 4. Conclusions

In this study, additive manufacturing was used to fabricate pads that fit human body shapes, and the fabricated pads were integrated into impact-protection pants. The curved 3D-mesh pads were designed for developing the impact protection pads with shapes adaptive to the human body’s surface, and its functionality was verified by presenting the results of the wearing sensory quality evaluation tests. We also used the body scan data for 3D modeling of the pads which allowed for the design of complex three-dimensional structures reflecting the curved surface of the human body in order to improve the fit and wearing comfort. A physical impact evaluation was performed on the developed protective clothing to verify the impact-absorption performance objectively. A subject group and an expert group evaluated the appearance, pad characteristics, motion functionality, and wearing characteristics of the protective clothing thereby verifying the practical use of the garment. The developed impact-protection pants were found to be suitable for the body shapes of elderly women and could be worn for a long time while performing daily-life activities. The protective pads reduced the impact by more than 82% and can help prevent hip fractures in the case of falls. This study confirmed the basis for the fabrication of new pads, which are unlike the existing fall-impact protection pads that use foam materials, using additive manufacturing. This study contributes to improving the quality of life for the elderly through the development of fall-impact protection clothing and can be extended to develop various types of protective clothing. In the near future, we plan to investigate the design and characteristics of a bridge connecting the hexagonal structure of pads for developing superior motion adaptable fall-impact protector using additive manufacturing.

## Figures and Tables

**Table 1:** Properties of the fabrics used in this study.

Properties	Main Fabrics	Lining (mesh)	Test Methods
Fiber content (%)	Polyester 51.1% (Thermolite^®^, Wilmington, DE, USA), Nylon 36.1% (Tactel^®^, Wichita, KS, USA), Polyurethane 12.8% (Lycra^®^, Wilmington, DE, USA)	Polyester 90.5%, Polyurethane 9.5%	KS K 0210
Weight (g/m^2^)	256.6	155.1	KS K 0514
Thickness (mm)	1.17	0.40	KS K ISO 5084
Stretch (%)	Warp 22.6	Wale 83.4	KS K 0352
	Weft 3.3	Course 94.5	

**Table 2:** Specifications of thermoplastic polyurethane (TPU) filament.

Properties	Value	Test Methods
Filament diameter	1.75 mm	-
Specific gravity	1.19 g/cc	ASTM D792
Tensile strength, yield	4 Mpa	ASTM D638
Tensile strength, ultimate	26 Mpa	ASTM D638
Tensile modulus	12 Mpa	ASTM D638
Elongation at yield	65%	ASTM D638
Elongation at break	660%	ASTM D638
Hardness	85 Shore A	ASTM D2240
Glass transition (Tg)	−35 °C	DSC
Melting point	216 °C	DSC

**Table 3:** Conditions of additive manufacturing.

Contents	Parameters	Values
Material	Flow	100%
Temperature	Extruder temperature	230 °C
	Bed temperature	40 °C
	Chamber temperature	40 °C
Quality	Layer height	0.2 mm
	Wall thickness	0.8 mm
	Bottom layer thickness	0.2 mm
Infill	Rate	100%
	Top layer count	6 each
	Bottom layer count	3 each
	Infill overlap	15%
Speed	Infill speed	40 m/s
	Inner wall speed	40 m/s
	Outer wall speed	30 m/s
	Bottom layer speed	20 m/s
	Travel speed	100 m/s

**Table 4:** Materials consumption of additive manufacturing.

Part	Material Consumption (m)	Weight (g)
Upper	34.56	103.92
Center	40.04	120.39
Lower	30.25	90.94
Side	10.44	31.39
Total	115.29	346.64

**Table 5:** Properties of CR foams.

Properties	Foam 1	Foam 2	Standard
Thickness (mm)	0.97	1.93	KS K ISO 5084
Hardness	23	25	ASTM D 2240
Density (g/cm^3^)	0.21	0.18	ASTM D 1056
Tensile Strength (Mpa)	1.3	0.8	ASTM D 1056
Elongation (%)	177	142	ASTM D 1056

**Table 6:** Body size of the subjects.

Contents	Subjects (n = 16)		60 Women (The 7th South Korea Human Size Survey)	
	Mean	SD ^1^^)^	Mean	SD ^1^^)^
Waist circumference (cm)	81.1	2.81	86.0	7.91
Hip circumference (cm)	93.0	2.61	92.6	5.13
Height (cm)	154.9	2.18	152.9	4.78
Weight (kg)	54.8	3.20	59.0	7.61

^1)^ Standard deviation.

**Table 7:** Results of appearance evaluation.

Contents	Subjects (*n* = 13)	Experts (*n* = 30)	*p*-Value
	Mean (SD)	Mean (SD)	
Is the color of the pants good?	5.00 (0.00)	4.40 (0.70)	0.009
Is the fabric of the pants good?	4.92 (0.28)	4.60 (0.52)	0.099
Is the fit of the pants good?	5.00 (0.00)	4.13 (0.82)	0.001
Is the design line at the part where the pad is inserted good?	4.92 (0.28)	4.27 (0.79)	0.014
Does the area where the pad is inserted look natural?	4.69 (0.48)	4.00 (1.08)	0.083
Is the location of the waistline suitable?	4.92 (0.28)	4.57 (0.73)	0.184
Is the waist circumference suitable?	4.77 (0.60)	4.50 (0.68)	0.243
Is the hip circumference suitable?	5.00 (0.00)	4.30 (0.88)	0.009
Does the crotch area appear good, without wrinkles/stretching?	4.85 (0.38)	4.07 (0.87)	0.006
Is the length of the pants suitable?	4.85 (0.38)	4.10 (1.16)	0.070

**Table 8:** Results of the evaluation of pad characteristics.

Contents	Subjects (*n* = 13)	Experts (*n* = 10)	*p*-Value
	Mean (SD ^1)^)	Mean (SD ^1)^)	
Is the location of the pad appropriate?	4.92 (0.28)	4.60 (0.70)	0.243
Is the shape of the pad appropriate?	4.85 (0.38)	4.40 (0.84)	0.159
Is the area of the pad appropriate?	4.62 (0.51)	4.40 (0.97)	0.784
Is the thickness of the pad appropriate?	4.69 (0.48)	3.90 (0.88)	0.004
Is the pad light?	4.38 (0.65)	4.00 (0.47)	0.074
Is the pad flexible?	4.77 (0.60)	4.30 (0.68)	0.032
Is the pad insertion method appropriate?	5.00 (0.00)	4.50 (0.71)	0.039
Is it easy to insert and remove the pads?	4.92 (0.28)	4.70 (0.48)	0.254

^1)^ Standard deviation.

**Table 9:** Results of the evaluation of motion functionality (*n* = 13).

Contents	Mean	SD
Are you comfortable when sitting in a chair?	4.92	0.28
Are you comfortable when squatting?	4.69	0.48
Is it comfortable to bend the upper body forward (90 degrees)?	5.00	0.00
Is it comfortable to bend your knees (90 degrees)?	4.62	0.51
Are you comfortable when walking?	4.77	0.44
Are you comfortable when going up and down the stairs?	4.77	0.44
Is it comfortable to make body motions, in general?	4.85	0.38
Cronbach’s alpha	0.62	

**Table 10:** Results of the evaluation of wearing characteristics (*n* = 13).

Item	Mean	SD
Is the feel of the pants good (outside)?	4.92	0.28
Is the feel of the pants good (inside)?	4.85	0.38
Is the stretch of the pants good?	4.85	0.38
Is it convenient to put on and take off the pants?	4.85	0.38
Is there any discomfort or pressure at the areas where the pads are inserted?	4.77	0.44
Cronbach’s alpha	0.61	

Standard deviation.
